# Revisiting starch degradability in dairy ruminants: from feed processing to gut microbiota and metabolic regulation

**DOI:** 10.1186/s40104-026-01365-3

**Published:** 2026-04-01

**Authors:** Qingyan Yin, Shengru Wu, Xinjian Lei, Jun Zhang, Dangdang Wang, Xinwei Li, Junhu Yao

**Affiliations:** 1 State Key Laboratory for Diagnosis and Treatment of Severe Zoonotic Infectious Diseases, Key Laboratory for Zoonosis Research of the Ministry of Education, Institute of Zoonosis, and College of Veterinary Medicine, Jilin University, Changchun, Jilin, 130062 P. R. China; 2 College of Animal Science and Technology, Northwest A&F University, Yangling, Shaanxi 712100 P. R. China; 3 National Center of Technology Innovation for Dairy, Hohhot, 010100 P. R. China

**Keywords:** Degradation sites, Energy efficiency, Feed processing, Ruminants, Starch

## Abstract

Starch serves as the primary energy source for high-producing dairy ruminants, which include both dairy cows and dairy goats. Optimizing starch digestion is crucial for ensuring high milk production and maintaining animal health. This narrative review summarizes and discusses recent findings concerning the degradability of starch in these species. Dietary starch is classified into three distinct types based on the basis of its degradation characteristics: rumen degradable starch (RDS), which ferments in the rumen; rumen escape starch (RES), which is subsequently digested in the small intestine; and resistant starch (RS), which resists complete digestion and enters the large intestine. This review systematically links feed processing methods, which directly influence starch structure, to their subsequent effects on the gut microbiota composition and host metabolic regulation. Three key insights emerge from this synthesis of literature. First, processing techniques such as steam-flaking critically alter the ratio among the three starch types, thereby shifting the effective site of digestion. Second, the optimal application of RDS differs significantly between dairy cows and dairy goats, primarily because these species exhibit distinct digestive physiologies. Nutritionists must carefully account for these species-specific differences to effectively prevent metabolic disorders. Third, the primary site of starch digestion significantly reshaped the gut microbiota profile. While a proper balance supports beneficial bacteria, excessive RS reduces energy efficiency, whereas an overload of RDS can readily lead to severe rumen acidosis. Therefore, balancing the proportions of RDS, RES, and RS is vital for helping animals effectively manage the elevated energy demands experienced during peak lactation. Future research must focus on developing precise starch management strategies tailored to the specific needs of various ruminant species.

## Introduction

Feed intake is essential for obtaining nutrients in animals. However, owing to shortages and high prices of protein, and the associated environmental impacts of protein production (e.g., land use, nitrogen waste, and greenhouse gas emissions), interest in low-protein, high-carbohydrate diets for intensive livestock production has increased [1–4]. Carbohydrates, as vital nutrients, provide energy to sustain the life activities of ruminants. The unique digestive system of ruminants enables them to consume and digest complex feed sources, including structural carbohydrates that are typically unsuitable for nonruminant diets. Carbohydrates can be classified into two categories: structural carbohydrates and nonstructural carbohydrates (NSCs), both of which play crucial roles in livestock production processes [5]. Among NSC, starch is the most important dietary component for ruminants, accounting for 15%–30% of their diet [6]. Starch undergoes rapid fermentation in the rumen, typically faster than dietary neutral detergent fibre (NDF). Understanding the degradation characteristics of starch in the gastrointestinal tract is vital for determining the appropriate amount of starch to be included in animal feed [7, 8].

In recent years, the concept of "precision nutrition" has emerged, emphasizing the need for optimizing dietary formulas beyond traditional parameters such as the concentrate-to-forage ratio. Achieving high milk yield and quality poses significant challenges, requiring in-depth studies and evaluations of effective nutritional indexes. Dietary carbohydrates, particularly their NSC fraction, are closely associated with milk yield and quality, whereas the structural carbohydrate fraction is essential for maintaining optimal rumen health and integrity. Notably, the type of starch and the ratio of branched (amylopectin) to linear (amylose) starch can significantly impact these factors [9]. As a major component of nonstructural carbohydrates, starch serves as a crucial energy source in ruminant diets. Considering the distinct utilization efficiencies of rumen-degradable starch (RDS) and rumen-escape starch (RES) in the rumen and intestinal tract, optimizing the dietary strategy by adjusting the functional ratio of RDS to RES may be more physiologically accurate [10, 11]. This represents a significant challenge for the dairy industry.

The objective of this review is to summarize recent research findings on the application of starch in high-producing dairy ruminants (dairy cows and dairy goats), with a focus on its effects on rumen and post-intestinal metabolism, animal health, and animal performance. We also discuss the key factors, specifically RDS, that determine digestive tract health and nutrient utilization in ruminants. In optimizing total mixed ration (TMR), it is essential to comprehensively consider the degradation rate and energy utilization efficiency of starch in different segments of the gastrointestinal tract, including the rumen and small intestine, in addition to dietary starch content.

## Role of starch in ruminant nutrition

Carbohydrates are essential nutrients in ruminant diets, and are derived from structural sources such as cellulose and hemicellulose, and nonstructural sources such as starch-rich crops (e.g., corn, barley, wheat, oats, and sorghum). Within the rumen, bacteria ferment available carbohydrates to produce volatile fatty acids (VFAs) and adenosine triphosphate. Starch is a major substrate for these processes, and its hydrolysis supports microbial growth and microbial protein synthesis [12, 13]. These monosaccharides serve as fermentable energy sources for rumen microbes and act as a primary source of glucogenic energy for high-yielding dairy ruminants [13]. Across the experimental literature, typical dietary starch inclusion for lactating dairy cattle is commonly reported in the range of 20%–30% of diet DM, with many controlled trials comparing “low” vs. “high” starch treatments roughly spanning 17%–31% DM [14–17]. For small ruminants (dairy goats/sheep), the reported dietary starch levels in experiments tend to overlap with those reported in cattle studies but can vary more (commonly 16%–35% DM) depending on the production system and diet formulation [18–21]. Some studies [14–17, 22–32] underscore the importance of optimizing starch levels and degradability to balance productivity and metabolic health in ruminants (Table 1). Available experimental evidence indicates that both the dietary starch level and the rate or site of starch degradation (RDS vs. RES) are closely linked to energy utilization and to the risk of ruminal and metabolic disturbances [33, 34].

**Table 1:** Typical starch inclusion in lactating ruminant rations and effects

Study	Species	Starch, % DM	Key effects (concise)
Dann et al. [14]	Cow	17.7–24.6	Lower starch-maintained milk yield and raised milk fat concentration
Dann et al. [15]	Cow	21.3–27	Replacing corn with fiber maintained fat corrected milk and improved rumen health
Hatew et al. [22]	Cow	11.0–21.7	High starch and fast fermentation altered volatile fatty acid and digestibility
Boerman et al. [23]	Cow	12.2 or 30.1	Higher-producing cows responded more negatively to a reduction in starch concentration than low-producing cows
Moate et al. [24]	Cow	24.0–30.0	Wheat (fast starch) reduced percentage and production of milk fat and ECM
Dias et al. [16]	Cow	23.0–29.0	Higher starch decreased rumen pH and total-tract digestion of organic matter
Sánchez-Duarte et al. [17]	Cow	21.0–27.0	Higher starch raised milk/ECM but tended to lower milk fat concentration
Xu et al. [25]	Dairy goat	RES 80–110 g/d	Optimal starch escape improved pancreatic enzyme secretion
Shen et al. [26]	Dairy goat	27.5–28.6	HRDS shifted hepatic lipid metabolism and raised lipopolysaccharide
Han et al. [27]	Dairy goat	27.5–28.6	HRDS disturbed hindgut microbiome, increased inflammation risk
Liang et al. [28]	Dairy goat	23.5 or 24.7	HRDS impaired growth and induced gut inflammation in kids
Chen et al. [29]	Dairy goat	28.4 or 54.3	High starch significantly increased duration in rumen pH and lipopolysaccharides
Lunesu et al. [30]	Sheep/Goat	7.8–20.0	Higher starch changed fermentation and gut histology
Zheng et al. [31]	Dairy goat	27.5–28.6	HRDS caused milk fat depression, metabolic and microbial disruption
Jin et al. [32]	Dairy goat	23.5 or 24.7	Starch overload caused hindgut dysbiosis and mucosal damage

*ECM* Energy-corrected milk, *VFA* Volatile fatty acid, *RDS* Rumen-degradable starch, *HRDS* High rumen-degradation-rate starch

### Starch requirements for maintenance and production

The energy requirements of ruminants vary with their physiological status. Previous studies have highlighted the importance of starch levels in the diets of 8 to 16-week-old calves [35]. In this review, we focus primarily on starch demand and utilization in high-producing ruminants (dairy cows and dairy goats), specifically during the peak lactation period. According to the NASEM (2021) [6], NSC intake is positively correlated with milk yield, highlighting its crucial role in supporting high milk production. Starch comprises a large share of nonstructural carbohydrates in crops such as corn, corn silage, and cereal grains (e.g., wheat or barley). Several common processing coproducts are also rich in starch; examples include broken rice, hominy feed, cassava (tapioca) meal, potato pulp, and bakery waste. For dairy cows, the acceptable proportion of dietary starch is typically approximately 25%, but it may be increased for high-yielding livestock [36, 37]. Cows at different stages of milk production respond differently to dietary starch intake. Evidence from a mid-lactation study in small ruminants showed that Saanen goats given higher-starch diets presented increased energy-corrected milk, which supports the idea that responses depend on the lactation stage [30, 38]. Therefore, the milk production level depends on the source of starch and its rumen degradation ratio. Increasing dietary RDS content in ruminant promotes fermentation of the ruminal propionate pathway, which facilitates efficient energy utilization [17]. However, excessively high rumen degradation rate starch (HRDS) can lead to abnormal fermentation, causing lactic acid accumulation [39] and the release of microbial products (lipopolysaccharide and histamine) that damage the rumen environment [40]. LPS is released from lysed Gram-negative bacteria and binds Toll-like receptor 4 (TLR4) on epithelial and immune cells, which activates NF-κB and MAPK signaling and triggers the release of proinflammatory cytokines (TNF-α, IL-1β, and IL-6); this pathway promotes rumenitis, increases epithelial permeability, and allows LPS translocation to the portal blood with downstream liver and systemic inflammation [39, 41]. Histamine is a biogenic amine formed by bacterial decarboxylation of histidine and signals via histamine receptors (H1–H4); histamine activates inflammatory pathways (including NF-κB and PKC/PKA cascades), alters vascular tone and permeability, and can reach peripheral tissues where it promotes local inflammation, impairs mammary protein synthesis, and contributes to the risk of laminitis [42]. Together, these metabolites compromise rumen barrier function and systemic health, and they help explain how severe fermentation shifts reduce production performance [43].

Feeding high-starch diets has been found to decrease the acetate-to-propionate ratio and reduce milk fat content and yield [44], particularly when the dietary content of rapidly degradable starch from the concentrate is high. These findings emphasize the importance of considering both the fermentative characteristics of the concentrate and its starch content when formulating diets to prevent subacute ruminal acidosis (SARA) [45]. Cows with different production levels clearly have distinct energy requirements. Thus, sorting cows on the basis of their productivity levels allows for more efficient feed allocation.

### Starch and rumen health

**Fig. 1:**
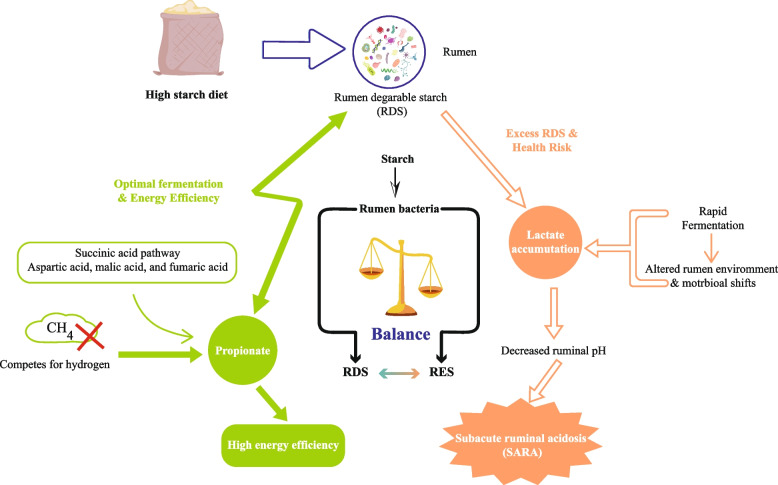
Effects of starch fermentation in the rumen impact on energy efficiency and health. RES, rumen escape starch

Rumen health is of paramount importance for overall ruminant health and productivity. Starch is initially transformed into VFAs within the rumen through the actions of rumen bacteria. VFAs serve as the primary energy source for ruminants, and propionic acid fermentation results in the highest energy efficiency [46]. High-starch diets promote propionate accumulation [47] while inhibiting methane production [48, 49]. Propionate can be produced through the succinic acid pathway, which uses dicarboxylic acids (e.g., aspartic acid, malic acid, and fumaric acid). This process competes for hydrogen with methane-producing bacteria, thereby reducing methane production and minimizing energy losses [50]. However, when the amount of RDS in the diet exceeds an animal's tolerance, it leads to increased VFAs and lactate production, resulting in decreased ruminal pH. This decline in ruminal pH is a major cause of SARA (Fig. 1).

Efficient rumen function is essential for overall ruminant health and productivity [51, 52]. High-yielding dairy cows often consume diets high in grain, which increases their energy supply but also poses a greater risk of SARA [53]. When the dietary starch concentration or the fraction of rapidly fermentable starch in the rumen increases, the ruminal VFA concentration (i.e., proportion of propionate) typically increases and gluconeogenic substrate availability improves; this can support milk and component yields in many situations [16, 17]. However, HRDS (especially from rapidly fermenting grains) can depress the ruminal pH, favor lactic acid accumulation and endotoxin release, alter the microbial community structure, and precipitate SARA and milk fat depression (MFD) in some herds [22, 26, 44]. Experimental comparisons therefore frequently show trade-offs: higher total starch (within physiological limits) can increase milk solid yields but increases the risk of ruminal disturbances if most of the starch is rapidly degraded in the rumen [23]. Starch fermentation in the rumen is intrinsically linked to environmental sustainability, specifically with respect to enteric methane emissions and nitrogen excretion. Increasing dietary starch generally shifts ruminal fermentation toward propionate production [54]. This shift is environmentally favourable because propionate formation acts as a hydrogen sink, competing with methanogens for metabolic hydrogen and thereby reducing CH_4_ production per unit of fermentable organic matter [55]. With respect to nitrogen metabolism, starch serves as the primary energy source for microbial protein synthesis. The synchronization between the RDS and rumen degradable protein is critical for optimizing nitrogen capture. Poor synchrony leads to uncoupled fermentation, where excess ruminal ammonia—unable to be incorporated into microbial protein due to a lack of energy—is absorbed and subsequently excreted as urinary urea nitrogen, a volatile form of nitrogen that contributes to environmental pollution [56]. Therefore, balancing starch degradability is essential for minimizing both the carbon and nitrogen footprints.

Some goat studies indicate tolerance to certain high-concentrate formulations, whereas other goat studies report clear rumen disturbance and MFD when RDS is very high [44]. Taken together, a practical implication from experimental evidence across high-producing dairy ruminants (cows and goats) is that when total dietary starch is increased (especially above 30% DM), reducing the fraction of RDS rapidly tends to reduce the risk of rumen acidosis and MFD and can preserve performance and health [17, 44, 46]. In other words, the site and rate of starch degradation (RDS vs. RES) are as important as the total starch concentration for balancing production and rumen/intestinal health. Balancing the energy needs of high-yielding dairy ruminants with maintaining normal rumen fermentation when formulating diets to prevent metabolic and digestive disorders is a critical challenge. Accurate determination of dietary starch content, considering both starch levels and degradation rates, is important for achieving optimal rumen metabolism and overall animal health.

### Starch and gut health

In ruminants, intestinal starch digestion follows a process that is generally similar to that in monogastric animals, in which starch escaping ruminal fermentation undergoes further enzymatic digestion in the small intestine by pancreatic amylase and is absorbed as glucose, which can yield more direct metabolizable energy than ruminal VFA under conditions of adequate small-intestinal digestion [57]; however, the small-intestinal capacity is limited and excessive rumen escape starch may reach the hindgut and cause digestive disorders, and pancreatic enzyme secretion increases with RES only up to a physiological plateau [58]. In line with this constraint, a study has shown that increasing RES induces a quadratic response in pancreatic α-amylase and lipase secretion, suggesting an adaptive but physiologically constrained pancreatic response [59]. Optimal production levels of α-amylase and lipase have been observed when RES is in the range of 80–110 g/d in adult goats [25]. Increasing α-amylase secretion in ruminants may increase postruminal starch digestion and thereby influence starch utilization efficiency in lactating dairy cows. The small intestine is the primary site for efficient nutrient absorption, and its absorption efficiency is regulated by various factors, including rumen digestion, digestive enzymes, and intestinal transport mechanisms. In lactating dairy cows, the contribution of the small intestine to starch utilization is often constrained by limited endogenous pancreatic α-amylase synthesis, and starch digestion in the small intestines of dairy cows is typically poor due to limited α-amylase synthesis. Increasing pancreatic α-amylase production has been proposed as a potential approach to improve the utilization of RES rather than total dietary starch. For example, studies have shown that duodenal infusion of leucine in heifers can increase the pancreatic α-amylase concentration and secretion as a nutrient signal [60] suggesting that leucine availability may link the amino acid supply to pancreatic exocrine function. In vitro studies have also indicated that leucine may function as a signaling molecule to regulate pancreatic α-amylase production by influencing proteasome activity and the mTOR pathway [61]; leucine regulates α-amylase and trypsin synthesis in dairy calves by raising mRNA expression and phosphorylation levels of protein components in the mammalian target of the rapamycin pathway [62], and leucine regulates α-amylase and trypsin synthesis in dairy calves by regulating the mTOR signaling pathways [63, 64]. Although these responses have been demonstrated mainly in calves or growing cattle, they provide a biological framework suggesting that leucine-mediated regulation of pancreatic α-amylase could influence RES utilization in lactating dairy cows, a hypothesis that still requires direct experimental validation.

The ruminant cecum is more developed than that of monogastric animals, as herbivores play a significant role in fermenting fibres and nonstarch polysaccharides. The large bowel is rich in microorganisms that ferment substances reaching this region. Resistant starch (RS), which refers to the portion of starch that escapes ruminal digestion and remains undigested before reaching the large intestine, serves as a substrate for fermentation by the microbial population, resulting in the production of VFAs. Recent studies have highlighted the role of dietary starch as a substrate for large bowel fermentation, emphasizing the physiological functions of VFAs [65]. Feeding RS has been shown to increase the production of total VFAs in the large bowel [66, 67]. However, exceeding the absorption capacity of VFAs in the hindgut, known as hindgut acidosis, can lead to the proliferation of pathogenic bacteria, a decrease in butyric acid levels, and the degradation of the mucin layer, ultimately compromising hindgut immune function and inflammation [27, 68, 69].

As the duration of the high-starch diet (32.34% of DM) increased, the elevated starch levels directly influenced the colonic lumen environment, subsequently impacting the composition and function of the microbiota specific to the colonic lumen. The colonic epithelium then gave rise to a new niche, which induces apoptosis in order to achieve functional transformation [70]. Furthermore, the effects of RS on the intestinal microbiota and its metabolites have been studied, highlighting the potential for RS to modulate the microbiota composition and function [71, 72]. The interaction between microbial communities and the host is crucial for adapting to dietary changes. Notably, other dietary components may also influence RS fermentation products [73].The balance of carbohydrates in the hindgut, particularly between starch and structural carbohydrates, is important for maintaining the health of ruminant animals.

## Mechanisms of energy supply from starch

**Fig. 2:**
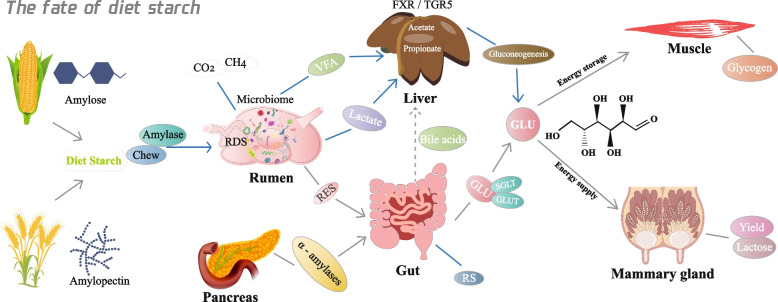
Dietary starch as a substrate for glucose supply in dairy animals. VFA, volatile fatty acid; RDS, rumen-degradable starch; RES, rumen escape starch; RS, resistant starch; GLU, glucose

After an animal ingests starch, rumen bacteria ferment the starch primarily into VFAs such as acetate, propionate, and butyrate, among which propionate plays a key role as the main precursor for glucose synthesis and the energy supply in ruminants. Any starch that escapes the rumen is digested in the small intestine, converted into glucose, and then directly absorbed [74]. However, studies have shown that small-intestinal digestion of RES in dairy cows is incomplete and variable, with reported digestion efficiencies typically ranging from approximately 50%–84% of RES, depending on the intake level, starch source, and physiological state [11]. While this intestinal glucose absorption provides a direct but quantitatively limited contribution to the animal’s energy supply, gluconeogenesis in the liver, which utilizes VFA (propionate) or amino acids, provides the majority of the glucose required by the animal [75]. In ruminants, hepatic gluconeogenesis supplies the majority of circulating glucose, accounting for approximately 75%–90% of total glucose entry into the bloodstream, whereas direct intestinal glucose absorption contributes only a minor proportion [76, 77]. Propionate is the dominant gluconeogenic precursor, providing approximately 60%–74% of the carbon used for hepatic glucose synthesis, with amino acids contributing [78]. Starch is a crucial energy source because it is the main dietary precursor for glucogenic substrates, especially propionate, which drives hepatic glucose synthesis (Fig. 2). Strategies for increasing the supply of these glucogenic nutrients include increasing the supply of intestinal digestible starch by using starch that has not been degraded by the rumen and increasing propionate production in the rumen via RDS [79, 80].

### Rumen degradable starch

The nutritional value of dietary starch extends beyond its mere content and includes the consideration of RDS. RDS takes into account the degradation and circulation rates of starch in the rumen, providing a more accurate indicator for evaluating the carbohydrate nutritional value in diet formulations. Feeding HRDS (24.88% of DM) diets to lactating goats has been associated with MFD, whereas low (20.52% of DM) and medium (22.15% of DM) RDS diets do not have this effect [44]. The onset of HRDS-induced MFD may be attributed to the promotion of hepatic lipid β-oxidation and disruptions in phospholipid and bile acid (BA) metabolism in the liver, consequently leading to a reduced supply of lipogenic precursors to the mammary gland in dairy goats [26]. Furthermore, the downregulation of genes involved in lipogenesis may contribute to MFD [44]. Thus, it becomes necessary to ensure animal health while simultaneously maintaining highly efficient energy utilization of starch by considering its degradation in the rumen. Increased levels of RDS significantly decrease the ruminal pH and increase the proportion of ruminal propionate, resulting in reduced production performance [44, 81]. Additionally, elevated RDS levels alter the gastrointestinal microbiota and fermentation, thereby affecting carbohydrate degradation and leading to decreases in the caecal amylose and cellulose contents. Therefore, comprehending the effects of RDS content on the gastrointestinal microbiota and the subsequent implications for animal health and production performance is crucial. Typical diets for high-yielding dairy cows show substantial variation in the proportion of RDS. For example, experimental dairy cow diets formulated with graded levels of RDS have reported rumen degradable fractions of 62% to 80% of total starch (LRDS 62.2%, MRDS 71.3%, HRDS 80.3% of total starch) [82]. In dairy goats, controlled feeding studies have defined low-, medium-, and high-RDS diets with RDS proportions of approximately 20.5%, 22.2%, and 24.9% of total starch, respectively, highlighting species-specific differences in diet formulation and rumen fermentation [44]. In sheep, variations in forage-to-RDS ratios likewise influence rumen fermentation characteristics, although direct quantification of RDS/RES proportions is less frequently reported [21]. Considering both starch degradation and the energy supply in the rumen, as well as exploring strategies to increase starch energy supply efficiency in the hindgut, is necessary, rather than solely focusing on rumen starch degradation.

### Rumen escape starch

In ruminants, starch can be classified as RDS, RES, or RS on the basis of its rate, extent, and site of digestion [17]. When grain-based diets are fed, up to 40% of dietary starch can escape ruminal fermentation and enter the small intestine for potential enzymatic digestion [83]. The direct supply of energy as glucose after efficient digestion and absorption in the small intestine is theoretically more efficient than ruminal fermentation and subsequent VFA production [83]. However, ruminants have a limited capacity for starch digestion in the small intestine; only 40% to 62% of RES is typically digested [84]. This low efficiency results in the waste of valuable feed resources. Starch depolymerization to release energy in the small intestine involves several enzymes, with α-amylase being the most important [85]. Hence, improving the digestion rate of starch in the small intestine to achieve efficient utilization throughout the intestine becomes a critical consideration. Like monogastric animals, ruminants depend on amylase enzymes secreted by the pancreas for small intestinal starch digestion [7]. Enhancing pancreatic amylase secretion or activity may represent an effective approach to improve small intestinal starch digestion [86].

### Methods for quantifying starch fractions and practical ranges

Accurate measurement of starch fractions—RDS, RES, and RS—is fundamental to optimizing ruminant diets, but it presents methodological challenges. The most common approach to estimate RDS is the in situ nylon bag technique, which provides fractional degradation rates under simulated rumen conditions. However, in situ methods face limitations, as they struggle to fully account for dynamic physiological factors in production settings, such as actual feed particle size reduction by chewing and rumination, and the variable passage rate of digesta. The RES is typically calculated as the difference between the total starch and the RDS fraction. The small intestinal digestibility of RES can be estimated via in vitro enzymatic methods or in vivo techniques such as the mobile-bag technique or total tract collection. These in vitro methods aim to simulate pancreatic α-amylase digestion. However, the in vitro results often struggle to fully reflect the true in vivo small intestinal digestion efficiency observed in the animal. The RS fraction, which escape both ruminal and small intestinal enzymatic digestion, is quantified primarily via sequential in vitro procedures involving heat-stable α-amylase and amyloglucosidase. In practice, the actual proportions of RDS and RES are highly variable, ranging typically from 50% to 90% for RDS and 10% to 50% for RES, depending on factors such as grain source (e.g., corn vs. barley), processing method (e.g., grinding vs. flaking), and feed intake level [6]. Generally, the higher the dietary starch content, the lower the level of RDS should be. Diet formulation relies on these ranges, often using predictive models that incorporate these in situ and in vitro data to estimate the actual starch flux through the gastrointestinal tract.

## Factors regulating starch digestion

The fate of dietary starch is highly variable and influenced by several factors, including starch type, processing methods, and interactions with other components of the diet [35, 87]. The degradation of starch in the rumen is regulated by the amount of daily starch intake, the rate of starch degradation, and the rumen meal circulation rate. Maintaining an optimal RDS content requires adjusting the grain content, selecting appropriate grain types, and employing suitable processing techniques during production. Various properties of starch, such as its crystal structure, particle size and shape, concentration of straight-chain and branch-chain starch, and protein matrix, influence its breakdown rate [88].

**Fig. 3:**
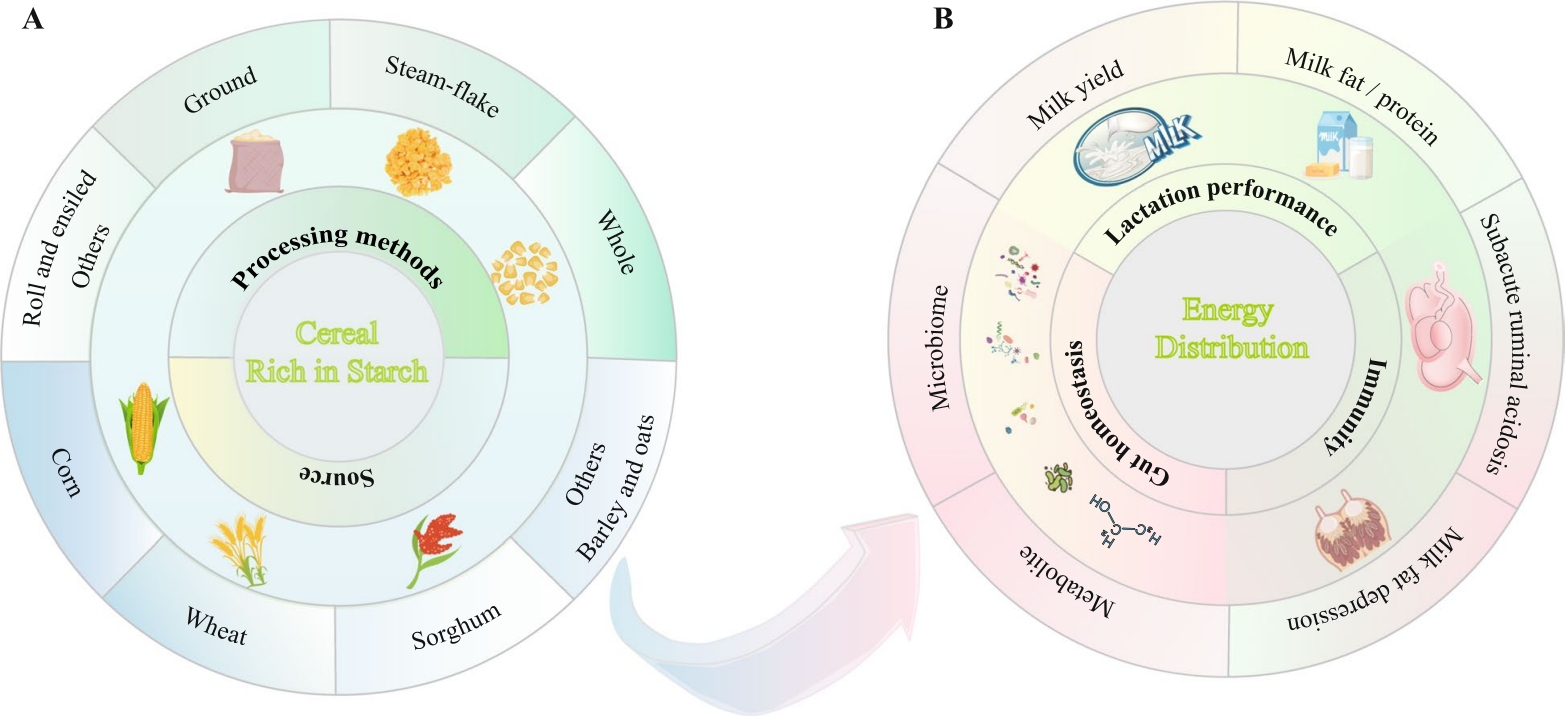
Influence of starch source and processing (**A**) on metabolic health and lactation efficiency (**B**) in dairy ruminants

The amylose content of starch is a key determinant of its physicochemical and functional properties [89]. Compared with corn, for example, barley and wheat, have a greater proportion of branched-chain starch and lower levels of insoluble protein, making them more susceptible to microbial interactions and leading to a higher rate of starch breakdown in the rumen [66]. Grain processing methods, including dry rolling, steam rolling, and steam flaking, are commonly employed to increase grain starch degradation in the rumen. These methods decrease the grain size, increase the surface area available for starch‒microbe interactions, and improve the breakdown rate. High-temperature steam treatment ruptures proteins and allows starch to be released, resulting in increased contact between starch and rumen microorganisms [90]. Overall, the physicochemical characteristics of starch and its processing methods collectively determine its ruminal degradability, thereby influencing nutrient availability, microbial fermentation patterns, and ultimately the productive efficiency of dairy ruminants (Fig. 3).

### Source of diet starch

Starch serves as the primary source of dietary energy for ruminants and is a crucial energy reserve in many plants. Common sources of dietary starch for ruminants include corn, sorghum, and barley. The starch content varies significantly among different plants, leading to variations in their ruminal degradation. Cereal grains, particularly corn, are rich in starch, which typically constitutes 70% to 80% of their DM content. However, geographical, genetic, and environmental factors contribute to the observed variation in starch composition among cereal grains [91]. Different forms of grain starch in ruminant diets, such as crushed or whole grains, and different sources of starch, such as wheat or corn, exhibit varying patterns of energy distribution after digestion in different sections of the gastrointestinal tract [25, 92, 93].

**Fig. 4:**
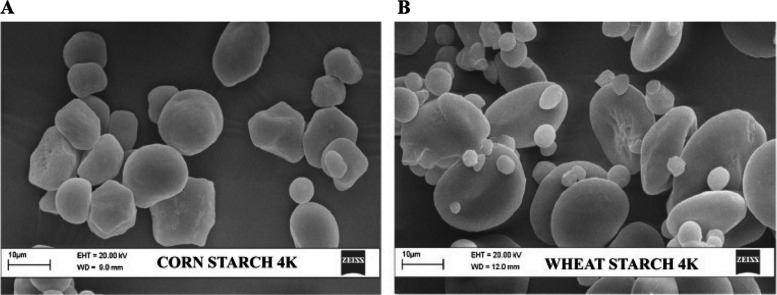
Particle size distribution of corn (**A**) and wheat (**B**) [94]

The physicochemical and functional properties of starch are determined by the particle size distribution and quantity of amylose and amylopectin, their ratio, and the internal structure of starch granules. For example, corn starch has polyhedral and round granules, and its particle size is approximately 5–18 μm. In contrast, wheat starch presented two distinct starch particle sizes: larger, type A (> 10 μm), and smaller, type B (< 10 μm) (Fig. 4). Type A granules display conventional irregular or spherical morphologies, whereas type B granules feature distinctive disk-like and lenticular shapes [94]. Starch pasting and digestion properties are also controlled to a significant extent by the chain length distribution of starch [91]. The corn amylopectin chain length distribution consists of short chains (DP 6–12) at approximately 24.0% and long chains (DP > 24) at approximately 23.5% [95]. The amylopectin chain length distribution in wheat varies, with short chains (DP 6–12) ranging between 44.5% and 52.4% and long chains (DP > 24) ranging between 3.7% and 6.5% [96]. These differences in starch composition between corn and wheat can impact their digestibility in animals. Substituting wheat for corn in dairy diets, even with no difference in starch content, can significantly reduce milk yield in lactating cows and dairy goats [97–99].

In reality, there are interactions between dietary components, and maintaining a balance between RDS and physically effective neutral detergent fibre (peNDF) is critical for maintaining appropriate rumen metabolism, as well as improving dairy cow output [100]. Moreover, studies have shown that SARA induced by feeding cracked wheat reduces NDF degradation in roughage and protein degradation in soybean meal (−19.8%) and extruded soy (−18.9%) while increasing starch degradability in corn due to the increase in the number of amylolytic bacteria and decrease in the number of cellulolytic bacteria in the rumen [101].

### Processing methods for cereals

The physicochemical properties of starch can be modified through physical methods, chemical processes, and genetic engineering [102]. The physical processing of corn, for example, provides greater net energy to cattle than does the use of whole corn [103]. Physical processing exposes the starch in the endosperm, altering its degradation in the animal's body. The separation and treatment of corn husk disrupt the crystal structure of starch, reducing its crystallinity. This treatment also breaks the hydrogen bonds between the crystalline and amorphous regions of starch particles, leading to increased water absorption, expansion of starch particles, and changes in the viscoelastic behavior of the starch slurry [104]. Consequently, starch degradation in the body of an animal is affected. Heat treatments can also influence the structural and physicochemical attributes of starch [105]. Chemical methods, such as enzymatic modifications, can increase the ratio of amylopectin to amylose under normal conditions [106]. *Staphylothermus marinus*, for example, exhibits high-temperature enzymatic activity that facilitates simultaneous gelatinization and hydrolysis of corn starch [107]. The inhibition of starch retrogradation can be achieved through treatment with 1,4-α-glucan branching enzyme, which increases the amylopectin content and reduces the amylose content [107]. Differences in corn starch morphology, technological properties, and thermal properties can be observed among different genotypes [108]. Corn, being a dominant and irreplaceable component of ruminant diets, undergoes different processing methods that affect its degradation and the absorption of other nutrients [109–112]. Consequently, nutritional management that balances starch source, degree of processing, particle size and physically effective fibre—often complemented by microbial modifiers (e.g., selected lactate-utilizing strains or yeast) or strategies to increase postruminal digestion—remains essential to steer the microbiome toward functional states that support both animal health and production.

### Species-specific considerations: cattle vs. small ruminants

While the fundamental principles of starch fermentation apply across ruminants, distinct physiological differences exist between dairy cattle and small ruminants (goats and sheep) that influence dietary formulation. Comparative studies indicate that small ruminants generally exhibit different digesta retention times and passage rates than lactating dairy cows do, particularly as feed intake increases. Compared with cattle, small ruminants have greater chewing efficiency and longer retention times relative to their body size, often leading to more extensive physical breakdown of grains and potentially HRDS in the rumen [113]. Consistent with this, feeding whole corn grain to small ruminants has been reported to stabilize ruminal pH, promote fiber degradation, and increase overall dietary starch degradability, likely due to enhanced mastication and gradual starch release in the rumen [19]. However, their total reticulorumen and hindgut capacities are significantly lower. This anatomical constraint means that goats and sheep have a smaller volume to buffer the rapid production of VFAs and lactate derived from high-starch diets. Consequently, small ruminants can be particularly sensitive to sudden increases in RDS, which may precipitate SARA more rapidly than in cattle. Furthermore, the limited capacity of the hindgut in small ruminants implies a reduced ability to manage large quantities of escape starch, potentially increasing the risk of hindgut acidosis when high levels of RES are fed. Therefore, extrapolation of starch limits from dairy cows to small ruminants requires caution, necessitating strict control of RDS levels to match their specific metabolic buffering capacities.

## Microbial and metabolic mechanisms governing dietary starch efficiency in ruminants

Rumen microorganisms, including *Streptococcus bovis*, *Ruminobacter amylophilus*, and *Prevotella bryantii*, produce a range of amylolytic enzymes (e.g., α-amylase and pullulanase) that catalyze the hydrolysis of starch into fermentable sugars, leading to the generation of VFAs [114], such as acetate, propionate, and butyrate. When the rate and extent of starch degradation are high in the rumen, total VFA production increases, providing key substrates for hepatic gluconeogenesis and increasing glucose availability. However, excessive fermentation decreases the ruminal pH, disrupts the microbial balance, and may impair fibre digestion efficiency. Conversely, when starch escapes ruminal degradation and reaches the small intestine, glucose derived from enzymatic hydrolysis by pancreatic amylase becomes a more efficient energy source because of increased postabsorptive conversion efficiency. Therefore, the efficiency of dietary starch utilization depends on the coordinated interaction between rumen microbial activity, fermentation end-product profiles, and host metabolic pathways.

### Microbial responses and functional adaptations to dietary starch

Microorganisms in the rumen play a crucial role in starch degradation, as the composition of the ruminal bacterial community is a major determinant of starch breakdown in addition to starch-specific parameters [115]. In any event, the vast majority of ingested starch is degraded in the rumen before it can fulfil its nutritious function [116]. Starch is primarily degraded in the rumen, although its energy production efficiency is lower than its breakdown in the small intestine. Nevertheless, the rumen serves as the primary site for starch breakdown, providing the majority of the energy supply for ruminants. Hence, regulating the rumen starch degradation rate or the rumen microbial environment is crucial for maintaining adequate energy supply and animal health. Microbial populations and nutrient availability interact closely in the rumen [117]. Excessive ruminal RDS degradation can lead to microbial shifts, such as decreases in the levels of GH9 family bacteria (Prevotellaceae, Ruminococcaceae, and Bacteroidaceae), which are responsible for cellulose degradation [26], and subsequently affect fatty acid hydrogenation [44].

Dietary starch exerts a primary and rapid selective pressure on the rumen microbiome by changing the available substrates [118], fermentation kinetics [9] and environmental pH [119]. Diets with moderate starch levels tend to favour a mixed community of amylolytic and fibrolytic organisms, whereas high starch intakes—particularly when supplied as HRDS from wheat or finely processed grains—promote the proliferation of amylolytic and lactic acid-producing taxa (e.g., Proteobacteria, *Megasphaera elsdenii*, *Streptococcus bovis*, *Selenomonas ruminantium*, and *Prevotella bryantii*). Concomitantly, the relative abundance and activity of classical fibrolytic taxa (e.g., *Butyrivibrio fibrisolvens* and *Fibrobacter succinogenes*) decline, as competition for surface-adsorbed substrates and low pH constrain cellulolytic enzyme expression and attachment [120]. Functionally, high ruminal starch drives shifts in carbohydrate-active enzyme profiles—upregulation of GH13-type α-amylases and starch-binding modules in amylolytic lineages, and downregulation of GH5/GH9 family cellulases—resulting in faster starch depolymerization but impaired fibre hydrolysis [81]. The microbial metabolic consequences include a redirection of fermentation end products toward propionate and lactate: propionate synthesis increases via the succinate and acrylate pathways (involving *Prevotella*, *Selenomonas* and *Megasphaera*) [120], whereas transient lactate accumulation may occur if lactate producers outpace lactate utilizers [121]. Such shifts reduce hydrogen availability to methanogens (often lowering CH₄ per unit of fermentable substrate) but at the cost of lowered ruminal pH and ecosystem stability [122].

**Fig. 5:**
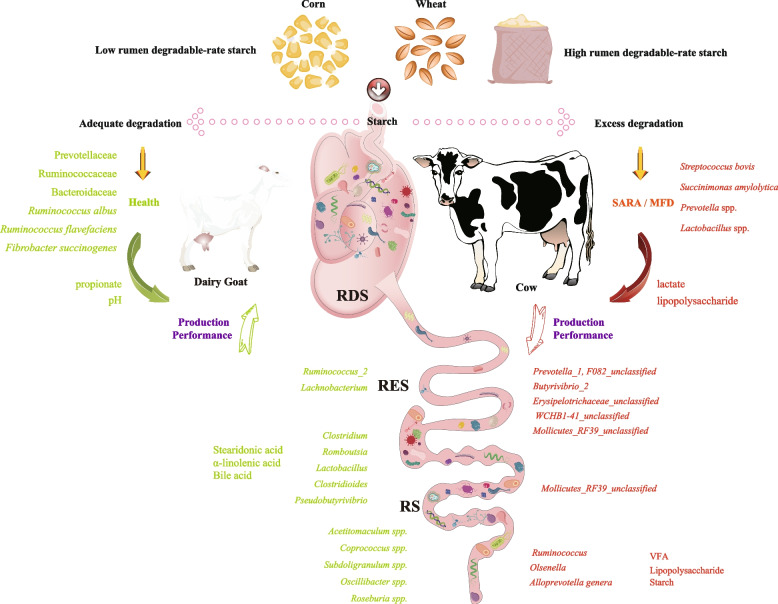
Dietary starch modulates gastrointestinal microbial and metabolite compositions in dairy cows and goats. MFD, milk fat depression; SARA, subacute ruminal acidosis; VFA, volatile fatty acid; RDS, rumen-degradable starch; RES, rumen escape starch; RS, resistant starch

The dietary starch content profoundly affects the gastrointestinal microbiota of dairy cows and goats, thereby shaping metabolite production and host physiology (Fig. 5). Under balanced conditions, moderate starch inclusion enhances populations of amylolytic bacteria such as *Streptococcus bovis* and *Ruminobacter amylophilus*, increasing the concentration of VFA, particularly propionate, which serves as the primary gluconeogenic precursor in the liver and directly supports milk lactose synthesis [81]. Acetate and butyrate, which are derived mainly from fibrolytic bacteria, including *Ruminococcus albus* and *Fibrobacter succinogenes*, contribute to milk fat synthesis through de novo lipogenesis in the mammary gland [123]. However, HRDS shifts the microbial composition toward rapid fermenters such as *S. bovis* and *Lactobacillus *spp., resulting in lactate accumulation, a drop in ruminal pH, and the suppression of fibrolytic taxa. This imbalance lowers NDF digestibility and increases the risk of MFD [124, 125], largely through changes in VFA ratios (acetate: propionate) and the accumulation of biohydrogenation intermediates that inhibit mammary SREBP1-mediated lipogenesis [44].

When starch escape to postruminal sites increases (either because of high dietary escape starch or ruminal overload), the small intestine and large bowel microbiota experience secondary disturbances with important health consequences. The small intestine of ruminants has limited enzymatic and absorptive capacity for starch; therefore, substantial RES may or may not be fully hydrolysed and absorbed, depending on pancreatic amylase secretion and particle accessibility [25, 58]. Undigested starch reaching the hindgut selectively enriches saccharolytic bacteria and increases VFA production in the cecum and colon, producing local drops in luminal pH and favouring acid-tolerant opportunists while impairing commensal fibrolytic populations [126]. Over time, this can reduce microbial diversity, alter the repertoire of CAZymes (fewer cellulases, and more amylases), increase the luminal LPS load from Gram-negative blooms, and degrade the mucous layer; histologically this manifests as epithelial inflammation, increased permeability, and in severe cases mucosal erosion [27]. These hindgut changes are particularly problematic in small ruminants (e.g., dairy goats), which have smaller gut volumes and thus less capacity to buffer acid fluxes and microbial perturbations, increasing their susceptibility to colonic dysbiosis and barrier dysfunction [32].

Given the central role of rumen microorganisms in starch fermentation, microbial interventions have been explored to redirect fermentation toward more stable outcomes. Supplementation with lactate-utilizing bacteria such as *Megasphaera elsdenii* has been shown to increase lactate clearance, mitigate postprandial pH decline, and support a more balanced VFA profile under high-starch feeding conditions in dairy cows [127–129]. Recent work has also indicated that combinations of *M. elsdenii* with yeast probiotics can further stabilize fermentation under acute acidosis challenges [130]. In addition, exogenous amylolytic or fibrolytic enzymes have been reported to alter starch degradation kinetics and improve total tract starch digestibility, although responses vary with diet composition, grain processing, and enzyme formulation [131–133]. These interventions, when aligned with diet design and microbial ecology, offer complementary tools for modulating ruminal starch fermentation without exacerbating the acid load.

### Metabolic mediators linking starch fermentation to host performance and health

The downstream physiological and production-level consequences of starch-driven microbiome remodelling are multifold and mechanistically interlinked. At the level of energy metabolism, increased ruminal propionate favours hepatic gluconeogenesis and thus can increase lactose synthesis and milk yield, but the concomitant reduction in acetate and butyrate availability deprives mammary lipogenesis of key precursors, often manifesting as MFD [44]. Microbial dysbiosis and a lower ruminal pH also impair fibre digestion and effective N utilization, reducing feed efficiency and sometimes increasing the amount of undigested nutrients in feces [134]. Systemically, barrier disruption and elevated LPS translocation activate innate immune responses (elevated acute-phase proteins, hepatic inflammation) and alter hepatic lipid metabolism [26]—responses that have been linked experimentally to the downregulation of lipogenic genes in the mammary gland and to shifts in BA signaling [31]. Finally, the risk–benefit trade-off between maximizing glucogenic flux (via propionate or intestinal glucose from RES) and maintaining microbial homeostasis emphasizes that not only total starch but also its site and rate of degradation (RDS vs. RES vs. RS) determine whether a given diet will improve productivity or precipitate metabolic and inflammatory disorders.

Moreover, the energy released from starch degradation supports rumen microbial protein synthesis. When transported to the liver, VFAs are utilized for gluconeogenesis, with propionic acid accounting for approximately 60%–74% and lactic acid accounting for 16%–26% of the produced glucose [135]. Additionally, VFAs, particularly acetic acid and butyric acid, have a significant effect on milk fat percentage, as they are involved in the synthesis of short-chain fatty acids and medium-chain fatty acids, which are crucial for milk production [136]. Given the central role of starch as an energy source, its degradation in the rumen to increase the VFA content and microbial protein synthesis or in the intestine to increase glucose metabolism significantly affects the energy supply and milk synthesis. Consequently, a high-starch diet is considered a key determinant of high milk yield. However, it is important to note that a rapid breakdown of starch in the rumen can pose a risk of SARA [134, 137–139]. Therefore, it is crucial to supply starch in a manner that balances production requirements with rumen health.

Microbial metabolites other than VFAs also play critical roles in mediating the starch–performance relationship. High-starch diets increase the levels of ruminal histamine and other biogenic amines produced via microbial amino acid decarboxylation (e.g., *Lactobacillus reuteri*), which impair epithelial integrity and activate inflammatory pathways via histamine H_2_ and H_4_ receptors, leading to rumenitis and systemic inflammatory responses [140]. Furthermore, Gram-negative bacterial lysis under starch-driven dysbiosis increases LPS release. Elevated LPS can cross a compromised epithelial barrier, enter the circulation, and activate TLR4 and NF-κB signaling in the liver, adipose tissue, and mammary gland, thereby stimulating proinflammatory cytokines (TNF-α and IL-6) and altering lipid metabolism. This mechanism contributes to hepatic lipid mobilization disorders and MFD [68, 141].

In addition, BAs represent an emerging microbiota-regulated signalling axis affected by starch supply. HRDS diets modify BA deconjugation and transformation via the gut microbiota (e.g., *Clostridium scindens* and *Ruminococcus gnavus*), shifting the balance of primary to secondary BAs. These altered BA pools modulate host metabolism through the activation of farnesoid X receptor (FXR) and Takeda G protein receptor 5 (TGR5). The activation of FXR downregulates hepatic lipogenesis via SHP-mediated inhibition of SREBP1c, whereas TGR5 signalling enhances energy expenditure and modulates glucose homeostasis [26, 27]. Thus, dysregulated BA metabolism under high-starch feeding not only reflects microbial shifts but also directly influences host lipid and energy metabolism.

HRDS-induced disruption results in stronger hindgut dysbiosis, elevated secondary BA accumulation, and intestinal inflammation, possibly due to species-specific differences in starch digestive capacity and microbial community resilience [44, 142]. Overall, dietary starch impacts production performance not only through VFA supply but also by modulating microbial-derived metabolites such as LPS, BA and histamine, which act through host receptors and signaling pathways to regulate milk synthesis, energy metabolism, and systemic inflammation. Optimal starch levels, coupled with controlled ruminal degradability and the rate of degradation, therefore represent a critical strategy for balancing energy efficiency and health in dairy production systems.

### Balancing starch-derived efficiency and host homeostasis

The modulation of starch fermentation represents a complex physiological trade-off. While the primary nutritional objective of increasing dietary starch is to maximize the glucogenic energy supply, this strategy operates on a precipice where the benefits of increased propionate production must be balanced against the risks of compromised microbial and epithelial homeostasis. Understanding this delicate equilibrium requires the integration of the functional roles of VFAs, LPS, and biogenic amines and the systemic inflammatory response. The glucogenic benefit of starch is physiological, and the synchronization of rapid starch degradation with microbial protein synthesis is the most effective driver of production efficiency. The fermentation of starch to propionate is thermodynamically favourable for the host, as propionate serves as the primary precursor for hepatic gluconeogenesis [143]. In high-producing dairy cows, this influx of propionate is critical for upregulating insulin signaling pathways that partition nutrients toward the mammary gland, thereby driving lactose synthesis and, consequently, milk volume [143]. Under optimal conditions, the rumen epithelium absorbs these VFAs efficiently, maintaining a pH amenable to cellulolytic function.

However, when the degradation rate of RDS exceeds the absorptive capacity of the rumen papillae, the accumulation of VFAs and lactate precipitates a decrease in the ruminal pH. This acidic environment acts as a potent stressor, triggering the lysis of Gram-negative bacteria, the subsequent release of cell wall components, specifically LPS, and the formation of biogenic amines such as histamine via the decarboxylation of histidine [144]. Concurrently, chronic exposure to low pH and high osmolality compromises the structural integrity of the rumen epithelium [145, 146]. The downregulation of tight junction proteins (e.g., claudin-4 and occludin) increases paracellular permeability—a phenomenon often described as "leaky gut" [147].

The translocation of LPS and histamine from the rumen into the portal circulation initiates a cascade of detrimental metabolic outcomes. Upon reaching the liver, these endotoxins stimulate Kupffer cells to release proinflammatory cytokines (TNF-α, IL-1 and IL-6), triggering an acute phase response [148]. This immune activation is metabolically expensive. It forces a repartitioning of nutrients: glucose and amino acids that would otherwise support milk protein synthesis are diverted to synthesize acute-phase proteins (e.g., haptoglobin, serum amyloid A) and support the oxidative burst of immune cells [148, 149]. Thus, despite the high energy density of starch-rich diets, the "net" energy available for production is reduced owing to this inflammatory tax.

Furthermore, this dysbiosis is intrinsically linked to MFD. The low pH environment favours alternative biohydrogenation pathways, leading to the accumulation of *trans*-10, *cis*-12 CLA, a potent inhibitor of mammary lipogenesis [123]. Recent evidence suggests that LPS exacerbates this effect by directly inhibiting lipoprotein lipase activity and suppressing the expression of genes involved in milk fat synthesis in the mammary gland [150]. Additionally, alterations in the enterohepatic circulation of BAs, induced by microbial shifts, may further impair lipid absorption and signaling, although this mechanism remains an emerging area of research compared with the established LPS-MFD axis. While maximizing starch fermentability offers a clear pathway to support the high glucose demands of lactation, it carries the inherent risk of triggering a "gut‒liver‒mammary" negative feedback loop. The excessive production of fermentation acids can initiate an inflammatory cascade that negates the energy benefits of the diet. Therefore, the ultimate goal of starch optimization is only to maximize degradation rates but also to define a threshold of fermentability that sustains gluconeogenesis without breaching the epithelial barrier, thereby preserving the intricate balance between metabolic fuel supply and host immunity.

## Future implications

The synthesis of evidence in this review underscores that starch is a double-edged sword: it is the most critical glucogenic precursor for high yield dairy ruminants, yet its fermentation represents the primary metabolic risk to host homeostasis. Optimizing ruminant performance requires not only maximizing starch intake but also precisely controlling the level and site of starch digestion, which is intrinsically mediated by microbiota‒host interactions. Despite significant advancements, several key knowledge gaps remain, particularly in defining and implementing optimal starch strategies. Future investigations must prioritize omics-based microbiome analyses and modelling. Future studies must move beyond basic bacterial community profiling. There is an urgent need for multi-omics approaches (metagenomics, metatranscriptomics, and metabolomics) to identify the specific microbial species and their gene pathways responsible for modulating the RDS/RES ratio and the production of specific toxic metabolites (LPS, histamine, and *trans*-fatty acids). This requires integrative systems modelling to link microbial functionality with host metabolic responses in a dynamic and temporal manner. Second, research must focus on the dynamic measurement of starch digestion kinetics. Current recommendations often rely on static feed analysis. Future research must develop noninvasive, dynamic measurement techniques to accurately quantify starch degradation throughout the entire digestive tract (i.e., the rumen, small intestine, and hindgut) in vivo. Establishing a suitable range of RDS and RES contents that maximizes the propionate supply without compromising epithelial integrity remains critical, with data lacking priority for different physiological stages of lactation. Third, the differences in starch sensitivity between cattle and small ruminants necessitate the development of refined species-specific and breed-specific models. Integrating breed-specific differences in passage rate and hindgut capacity is essential to translate general starch guidelines into precise recommendations that mitigate breed-associated risks of acidosis. Finally, the goal is to integrate precision nutrition strategies. Future work should focus on the use of dietary factors (e.g., specific grain processing methods and targeted feed additives such as essential oils or amino acids) to predictably modulate the rumen environment, thereby facilitating efficient starch degradation and regulating the distribution of starch absorption (VFA vs. glucose) to sustainably improve the health and production of high-yielding ruminants.

## Data Availability

No datasets were generated or analysed during the current study.

## Abbreviations

BA: Bile acid
FXR: Farnesoid X receptor
HRDS: High rumen-degradation-rate starch
LPS: Lipopolysaccharide
MFD: Milk fat depression
NDF: Neutral detergent fibre
NSC: Nonstructural carbohydrates
peNDF: Physically effective neutral detergent fibre
RDS: Rumen degradable starch
RES: Rumen escape starch
RS: Resistant starch
SARA: Subacute ruminal acidosis
TGR5: Takeda G-protein receptor 5
TMR: Total mixed ration
VFA: Volatile fatty acid
